# Movement-Contingent Time Flow in Virtual Reality Causes Temporal Recalibration

**DOI:** 10.1038/s41598-019-40870-6

**Published:** 2019-03-13

**Authors:** Ambika Bansal, Séamas Weech, Michael Barnett-Cowan

**Affiliations:** Department of Kinesiology, University of Waterloo, Waterloo, ON N2L 3G1 Canada

## Abstract

Virtual reality (VR) provides a valuable research tool for studying what occurs when sensorimotor feedback loops are manipulated. Here we measured whether exposure to a novel temporal relationship between action and sensory reaction in VR causes recalibration of time perception. We asked 31 participants to perform time perception tasks where the interval of a moving probe was reproduced using continuous or discrete motor methods. These time perception tasks were completed pre- and post-exposure to dynamic VR content in a block-counterbalanced order. One group of participants experienced a standard VR task (“normal-time”), while another group had their real-world movements coupled to the flow of time in the virtual space (“movement contingent time-flow; MCTF”). We expected this novel action-perception relationship to affect continuous motor time perception performance, but not discrete motor time perception. The results indicated duration-dependent recalibration specific to a motor task involving continuous movement such that the probe intervals were under-estimated by approximately 15% following exposure to VR with the MCTF manipulation. Control tasks in VR and non-VR settings produced similar results to those of the normal-time VR group, confirming the specificity of the MCTF manipulation. The findings provide valuable insights into the potential impact of VR on sensorimotor recalibration. Understanding this process will be valuable for the development and implementation of rehabilitation practices.

## Introduction

The ability to estimate the passage of time with precision is fundamental to our ability to perceive and interact with the world. One key characteristic of time perception is that it is highly plastic, supporting the ability to adapt to changing environmental conditions^1–4^. Although the plasticity of time perception has been well established, several open questions remain regarding the strength, persistence, and specificity of temporal recalibration effects. For instance, do these recalibration effects generalize across different modes of duration estimation (e.g., continuous and discrete motor reproduction)? Answering such questions will improve the understanding of the computational and neural basis for time perception, which has gained significant interest of late partially due to the appeal of preventing or reversing maladaptive changes to time perception with age^5–8^.

## Computational Perspectives of Time Perception

Time perception is a multifaceted construct, and several theoretical perspectives have been proposed in the temporal processing literature (for review, see^9^). The dominant model of temporal processing involves a central internal clock^10–13^. In this model, time perception is represented by the subjective count of pulses that accumulate within a given interval. There is evidence^14^ that these mechanisms, theorised to underlie the conscious perception of time, also drive the timing of motor performance (also see^15^). Research suggests that the neural pacing signal of the internal clock system, rather than being static, can be modulated by sensory inputs from external stimuli. A supra-modal system contrasts with the idea of dedicated mechanisms for each sense, although it is possible that a central mechanism subserves supra-second intervals, while sub-second intervals are processed by modality-specific systems^12,16^. Others have proposed models that do not require pulse intervals for timekeeping, suggesting instead that time is encoded in spatial^17,18^ or temporal^19^ patterns of neuronal firing. These state-dependent network models have gained recognition on the basis of support from psychophysical studies of temporal processing and learning mechanisms^20–22^. While each model has proven useful, there is a lack of compelling *in vivo* physiological evidence for the existence of a pacing signal or state-dependent representations of timing^23^.

## Neurophysiology of Time Perception

Describing the processes underlying time perception at the neural level has been a major challenge in the field. Although still a matter of debate, the brain structures thought to be involved in time perception include the cerebellum, prefrontal cortex, basal ganglia, and supplementary motor area^16,24,25^. Recent transcranial magnetic stimulation (TMS) evidence has supported the role of the cerebellum and prefrontal cortex in time perception. TMS used to disrupt cerebellar activity impairs timing of sub-second durations^26–28^, whereas inhibitory TMS over prefrontal cortex impairs timing of supra-second durations^27,29^. It has been proposed that the prefrontal cortex operates in a feedback role by using the sensory information following an action to update temporal expectations^30^, whereas the cerebellum plays a feed-forward role in making temporal predictions prior to an action^16^. Basal ganglia activity is associated with the encoding of temporal processing and representation of stimulus duration, which is demonstrated by both the behavioural data of Parkinson’s patients with basal ganglia dysfunction^31^ and by functional magnetic resonance imaging (fMRI) studies^24,32–34^.

These neuroanatomical studies in conjunction with non-human pharmacological studies have supported the idea that timekeeping is modulated by dopamine neurotransmission, specifically at the D2 receptor^16,35,36^. Dopamine antagonists (e.g., neuroleptics) decrease subjective estimations of event duration^37^, whereas dopamine agonists (e.g., methamphetamine) lead to an increase in duration estimations^38,39^. Recent evidence also suggests a prominent role for GABA in time perception; magnetic resonance imaging of the rat cortex shows that elevated GABA levels correspond to underestimation in the perceived duration of sub-second intervals, possibly due to diminished awareness of visual stimuli^37,40^.

## Evidence of Temporal Recalibration

Neurophysiology studies not only provide insight into the neural mechanism of the internal clock, but they also support the idea that time perception can be manipulated. The speed of this internal clock can be increased or decreased depending on the drug administered, which can lead to behavioural changes^38,41^. Another phenomenon that has been shown to speed up the internal clock is the click train effect, whereby listening to a train of clicks (e.g., 5 sec of clicks at 5 clicks/sec) induces a 10% decrease in the perceived duration of subsequent intervals^2,14,42^. In line with pharmacological studies, the click train is thought to speed up the internal clock by increasing arousal levels acting on the calibration unit. The stimulus preceded by the train of clicks is therefore perceived as shorter than the one preceded by silence. These studies provide evidence that temporal recalibration can occur, at least at the sub-second scale. Additionally, evidence shows that motor reproduction of interval timing is similarly affected by click trains, suggesting that a common temporal oscillator may underlie both conscious time perception and motor performance^14,15^.

Changes in temporal processing have been reported following repeated exposure to temporal misalignments in multimodal cues, in the form of a shift in the estimated simultaneity of post-training stimuli^4,43–45^. Temporal recalibration effects in response to novel temporal correlations between motor performance and sensory feedback have also been described. Latency between action and visual feedback leads to predictable and persistent behavioral adaptation aftereffects^3,46–48^. Rohde and colleagues^3^ observed perceptual learning effects when a lag was introduced between hand and cursor movement in a manual tracking task. Adaptation to visuo-motor latency revealed a decrease in motor error with time, and large aftereffects in motor timing following adaptation. As in previous studies^47,48^, the recalibration effect was found to generalize between motor timing and perceptual measures (e.g., simultaneity judgments).

## Perceptual learning as a Framework for Temporal Recalibration

Most studies that have examined the plasticity of time perception have adopted a perceptual learning approach. Theories such as sensorimotor contingency theory^49,50^ highlight the role of the action-perception loop in achieving perceptual learning. This theory describes sensorimotor contingencies as the role our actions play on the sensory inputs we receive: perceptual qualia emerge from learned relationships between action and the incoming sensory data produced as a consequence of the action. These sensorimotor contingencies are implicitly learned over time and shape perception^51^. Bompas and O’Regan^52^ provide an elegant example of how sensorimotor contingencies are learned by artificially coupling eye movement and color changes. By exposing participants to specific colors upon saccades to the right or left over an extended duration, they produced a predictable and persistent change in the perceived color of a neutral patch that was contingent on saccade direction.

If the qualia of perception are determined by environmental interaction, it is conceivable that even high-level perceptual qualia such as time can be altered by inducing a novel sensorimotor contingency

## Applications of Plasticity in Time Perception

Manipulations of time perception are of practical interest. Several neurological conditions are associated with deficits in the perception of timing, including ADHD^53^, autism^54^, and schizophrenia^55^, and these deficits can be reduced through timekeeping training^56,57^ (for review, see^58^). This decrement in time perception has also been seen in the normal aging process^6,8^. Due to the appeal of preventing or reversing these maladaptive changes, the utility of interventions that influence time perception has been widely sought after. Although several studies have investigated the plasticity of time perception in the framework of perceptual learning, studies have not examined whether exposure to a novel relationship between action (e.g., moving the body) and sensory feedback (e.g., the speed and duration of environmental events) can produce changes in time perception at the supra-second time-scale.

## Study Objectives

The objective of this study was to investigate if temporal recalibration is induced by exposure to a novel sensorimotor contingency between movement and the speed of events. This question can only be investigated using a naturalistic setting that affords re-learning of normal action-perception loops. In order to provide a naturalistic setting in a controlled environment, we chose to administer the movement-contingent task using virtual reality (VR) technology. VR, which uses sensory stimulation devices to simulate an interactive environment, has the unique ability to dissociate the natural link between perception and action^59^.

We attempted to induce a novel sensorimotor contingency by coupling the speed and duration of visual events to the bodily movement of the participant. We term this ‘movement-contingent time-flow’ (MCTF). In this manipulation, if the participant moved their hands or head, the speed of events in their surrounding virtual environment was normal. However, if the participant stopped moving, the speed of events slowed down. With exposure to this manipulation, we expected participants to adapt their perception of time such that when they were static, the probe durations were perceived as longer. To test if temporal recalibration had occurred, we conducted pre- and post-exposure time perception tasks. The psychophysical tasks required participants to observe a probe moving in a circle and then reproduce the duration, speed, and trajectory of the probe. We assessed both continuous motor and discrete motor time perception tasks. We also measured the effects of VR without the MCTF mechanic, to assess if exposure to VR alone affected time perception. As an additional control, we conducted a non-VR control task for a subset of participants in order to determine whether physical activity alone results in temporal recalibration.

## Hypothesis

The present study tests the hypothesis that adaptation to a novel relationship between action and the perceived timing of events in virtual reality results in recalibration of time perception.

We predicted time would be perceived as slower when participants were static following exposure to VR with the MCTF manipulation, due to the novel relationship between movement and event speed acquired during the task. As such, we predicted different pattern of data across the continuous motor and discrete motor tasks. For the continuous motor task, participants were static when observing the probe, and were moving when reproducing the probe. Perceived durations were predicted to be longer when the probe was observed (no movement), and shorter when the probe was reproduced (movement). This difference was not predicted for the discrete motor task, where the ‘observe’ and ‘reproduce’ phases of the trials were both static (see Table 1).

**Table 1:** Predictions for continuous motor and discrete motor time perception tasks following adaptation to VR movement contingent time-flow.

	Observe Probe	Reproduce Probe	Predicted difference (Reproduce minus Observe)
Continuous Motor task	Static Participant (perceived as ‘long’ durations)	Dynamic Participant (perceived as ‘short’ durations)	Negative timing difference (‘short’ minus ‘long’ durations)
Discrete motor task	Static Participant (perceived as ‘long’ durations)	Static Participant (perceived as ‘long’ durations)	No difference (‘long’ minus ‘long’ durations)

We expected to find no difference between pre- and post-adaptation estimates for participants who were exposed to VR with no MCTF manipulation, or by participants who simply performed a dynamic motor coordination task (a ball-toss) instead of the VR task.

## Methods

### Participants

Thirty-four students from the University of Waterloo participated in the study (the final dataset included data from 31 participants due to our exclusion criteria, as described in Results; 18 females, 13 males; age in years *M* = 21.1, *SD* = 2.4). All participants reported having normal or corrected-to-normal vision and reported no sensory, musculoskeletal, or neurological disorders. Protocols were approved by the University of Waterloo Research Ethics Committee and were carried out in accordance with the Declaration of Helsinki. All participants gave informed written consent, but all were naïve to the hypotheses of the research. Participants either volunteered their time or were remunerated $10 per hour for their participation. With respect to participants whose data comprised our final dataset, prior experience with VR was generally low – nine participants had used VR before, but all of those had only used it on one occasion. In addition, with respect to average experience with video games, the final dataset consisted of the following distribution of participants: 1 reported playing ≥15 hours of video games/week, 3 reported playing 5–14 hours per week, 9 reported playing video games for ≤5 hours/week, and 18 participants reported that they typically did not play video games.

### Apparatus

The equipment used for the time perception tasks was a laptop (R590, Samsung, 1366 × 768 resolution, 60 Hz refresh rate). The laptop was positioned at the participant’s eye level and the approximate distance from the screen to their eyes was 60 cm (visual angle was ~18 × 32**°**), although a chin rest was not used. The time perception task was run using Matlab R2016a with the Psychophysics toolbox^60^. The mouse used in this task was a USB mouse (Apple Inc.), which was positioned on a mouse mat with a gel wrist rest.

The VR environment was presented via a head mounted display (Rift CV1, Oculus VR; 90 Hz refresh rate, 1080 × 1200 resolution per eye) with hand-held controllers (Touch, Oculus VR). The headset and controllers were motion tracked by a combination of the inertial (accelerometer/gyroscope) and optical (3 x infrared Oculus cameras) sensors that were included with the system. Movement of the head was translated into motion of the observer viewpoint in the VR task. The system ran on a custom built workstation with a high-end graphics board (GTX 1070, NVIDIA). The software packaged with the head mounted display was used to calibrate the capture space (2.41 × 2.41 m) and the inter-pupillary distance of the headset for each participant.

The equipment used for the non-VR control task included 20 foam-rubber balls, three buckets, and a pair of tight fitting goggles. The foam-rubber balls were 5.08 cm in diameter and consisted of both white and colored balls. The target buckets were 21.59 cm wide and 21.59 cm tall. The goggles, which were used in order to replicate the narrow visual field of the head-mounted display, produced a field-of-view of approximately 90 × 50**°** (horizontal x vertical).

#### Time perception tasks

**Figure 1:**
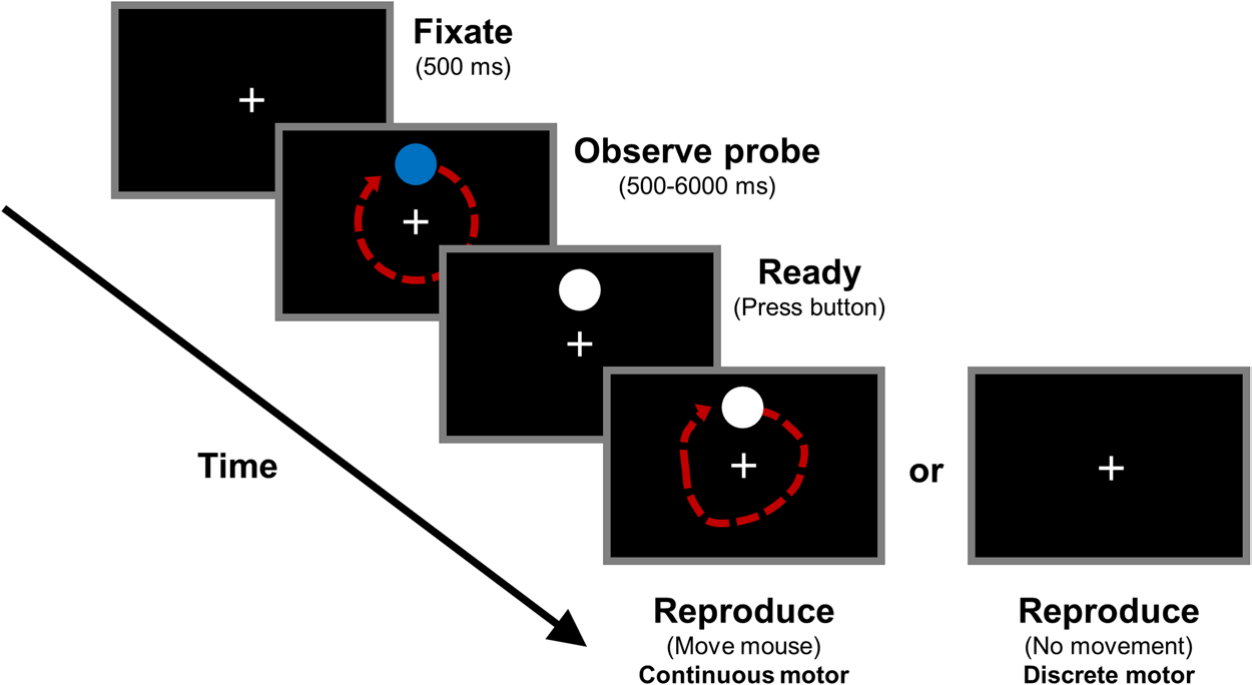
Progression of the continuous motor and discrete motor reproduction time perception tasks.

In the time perception tasks, participants were first presented with a fixation cross for 500 ms. Participants were instructed to fixate on the cross throughout the task. Participants were presented with a blue circular probe located at approximately 3.5**°** eccentricity from the central fixation cross. The probe then rotated around the fixation cross for a given duration and velocity before disappearing. Next, participants were prompted to reproduce the timing of the probe using one of two methods. The first method required continuous motor reproduction of the probe’s spatiotemporal trajectory by moving a white circle using a mouse (the continuous motor task), and the second method required a button press to signal the start and end of the perceived probe duration (the discrete motor task). All responses were recorded at the moment of button-press (i.e., not on button-release). The two tasks are described in detail below (also see Fig. 1).

Continuous motor reproduction task: To reproduce the speed and duration of the probe movement, participants were instructed to move their hand in a circle while holding a mouse. The white circle that appeared after the probe disappeared moved in a one-to-one manner relative to the hand movement of the observer. Participants were required to press the mouse button to indicate duration onset and offset. Supplementary Movie 1 depicts four example trials from this condition.

Discrete motor reproduction task: To reproduce the duration of the probe movement, participants first pressed the mouse button indicating duration onset, then waited until the perceived duration had elapsed, and finally pressed the button again to indicate duration offset.

#### Virtual reality tasks

**Figure 2:**
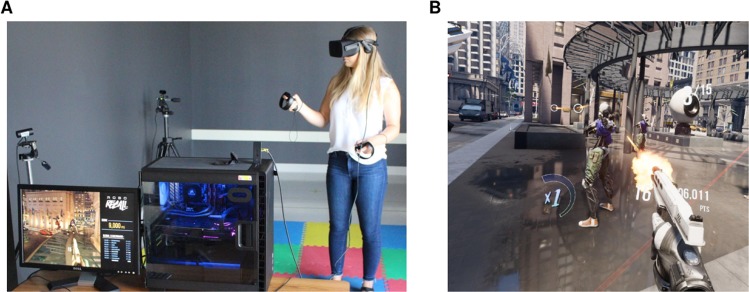
(**A**) Depiction of the setup used for the VR task. (**B**) Screenshot of the VR content (Robo Recall, Epic Games, NC, USA).

The VR content used in the experiment was an off-the-shelf consumer game (Robo Recall, Epic Games; Fig. 2). This was a first-person action game wherein the user took the role of a robot whose task was to destroy other robots that had been let loose in a realistic city environment. Participants were instructed to play the game by shooting the target robots until the time limit was reached. Points towards the participants’ scores were received upon performing one of several actions, such as destroying an enemy, avoiding enemy projectiles, and chaining together multiple enemy takedowns. All points were computed by the in-game scoring system and the experimenter recorded the total score after termination of the VR block.

Depending on their group assignment, participants experienced one of two versions of this game. The manipulated factor between these groups was whether the speed of events in the game was either normal (VR control, *n* = 13 following exclusions: see results) or modified (VR MCTF, *n* = 12 following exclusions: see results) as a function of the movement of the player’s hands (tracked by the Touch Controllers) and head (tracked by the Oculus Rift headset). In order to achieve the latter, we implemented a user-made modification of the game (MGS Studios, Berkshire, UK). In this modification, the movement of the participants was coupled to the speed of events occurring in their surroundings. If the head and hands were stationary, event speed was decreased by a factor of 8 compared to control conditions (e.g., a ball that would normally hit the ground after 1 s of falling would instead take 8 s to fall). Conversely, movements of 100 cm/s or greater were associated with normal event speed (i.e., no decrease in the speed of events). Participant movement speeds between 0 cm/s and 100 cm/s were linearly and inversely related to event speed; for example, 70 cm/s movement caused event speed to decrease by a factor of 2.4 (30% of 8), and 30 cm/s movement reduced event speed by a factor of 5.6 (70% of 8). The program did not combine the speed of multiple sensors (e.g., moving both hands at 40 cm/s resulted only in a factor of 4 decrease in event speed). In the VR control condition, participants were exposed to the same experience, but without the movement-time coupling.

#### Non-virtual reality control task

In addition to the two VR groups (MCTF and control), we conducted a non-VR control task with a subset of participants (*n* = 6) in order to decouple the effect of VR exposure from the possible effects of physical activity on time perception, as durations tend to be overestimated following physical activity^61,62^.

**Figure 3:**
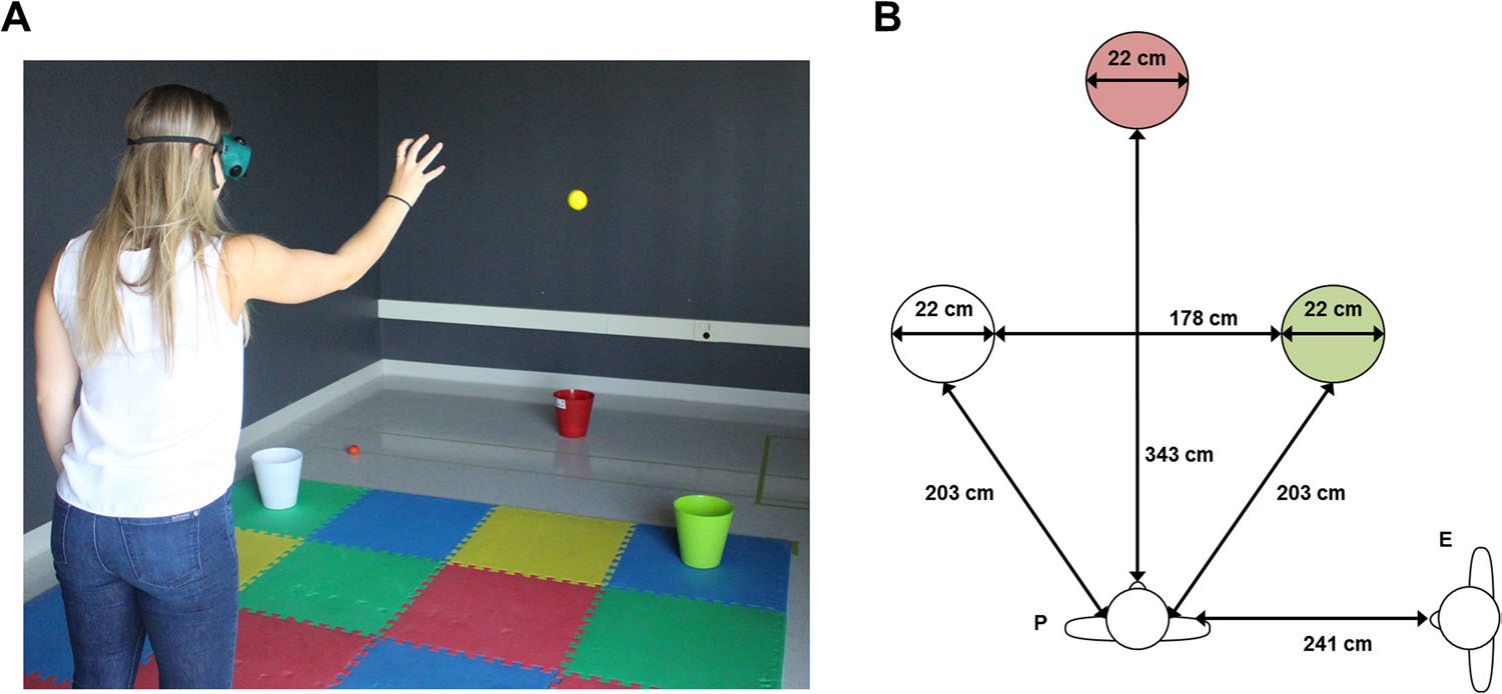
(**A**) Depiction of the non-VR control task. (**B**) Schematic overview of the non-VR control task from a bird’s-eye view. P = Participant; E = Experimenter. Objects not presented to-scale.

The objective of the task was to throw foam balls into the correct color of bucket in order to accrue points (Fig. 3). The experimenter began the task by throwing the balls one at a time to the participant, who was instructed to toss the white balls into the white bucket, and the colored balls into the green bucket (correctly doing so gained the participant 1 point). Participants were also informed that they could throw any of the balls into the red bucket, thus earning 4 points. Participants were instructed to accrue the highest possible points tally. Once all of the balls in the bag were tossed, participants walked around the area to collect the balls that did not reach the buckets, while the experimenter counted the ones that did. Once the balls were returned to the experimenter, the next round of throws commenced. This process continued until 10 minutes had elapsed.

### Design and procedure

Participants first completed two time perception tasks (continuous motor and discrete motor) to establish pre-adaptation measures (details below). Next, participants were exposed to the VR task and a post-adaptation time perception task (e.g., continuous motor). Finally, participants were exposed to the VR task a second time and then completed another post-adaptation time perception task (e.g., discrete motor). Participants each had a predetermined group assignment and order of time perception tasks that were counterbalanced across participants.

Each time perception task contained 15 unique trials, which consisted of 3 angular velocities (25, 75, 125**°/**s), and 5 different durations that spanned a logarithmic space (0.5, 0.9, 1.7, 3.2, 6.0 seconds). Each trial was repeated 5 times, resulting in a total of 75 trials per task. The order of the trials was randomized. The order in which the continuous motor and discrete tasks were performed was counterbalanced across participants. Each time perception task required approximately 10 minutes to complete. Upon performing the time perception tasks for the first time (pre-adaptation), 4 practice trials were completed. The 4 practice trials were conducted at 0.5 and 6 seconds for both 25 and 125**°/**s.

Participants were randomly assigned to one of the two VR conditions: MCTF VR or control VR. In both conditions, the VR exposure occurred in two phases and exposure lasted for 10 minutes in each phase. An additional group of participants were assigned to complete the non-VR control task.

## Results

We assessed if participants correctly performed the time perception tasks by measuring the correlation between the ‘actual’ and ‘perceived’ durations across trials for each individual (conditions and trial blocks were pooled). The data from one participant were excluded from further analysis due to a non-significant correlation (*p* > 0.05), and data from two additional participants were excluded due to errors with data recording (excluded from VR MCTF *n* = 2; excluded from VR control *n* = 1; none excluded from non-VR control). For the remaining 31 participants the mean Pearson *r*(148) score for the correlation between actual and perceived durations was high, both in the continuous motor task (*M* = 0.89 ± 0.09 *SD*), and in the discrete motor task (*M* = 0.89 ± 0.08 *SD*; all *p*s < 0.001).

Analysis of participants’ performance in the VR task revealed that there was no significant difference between scores in the first and second VR blocks (paired samples *t*-tests; VR Control, *t*(12) = 1.31, *p* = 0.21; VR MCTF, *t*(11) = 0.81, *p* = 0.43), nor was there a difference between the VR MCTF and VR Control groups (independent samples *t*-tests, block 1, *t*(23) = 1.27, *p* = 0.22; block 2, *t*(23) = 1.85, *p* = 0.08).

Informal observation of participants during the task indicated that participants in the VR MCTF group used several strategies in order to optimise performance in the task. The majority of participants demonstrated a ‘freezing’ strategy during the task: the participant would adopt and maintain a specific posture for 2–3 seconds and observe the environment while time was moving slowly; following this, the participant would break out of this posture in order to act (e.g., shooting an enemy). While the majority of participants in the VR MCTF group adopted this strategy, it was used infrequently, and did not appear to result in measurable differences in performance (i.e., total point scores).

### Main effects

**Figure 4:**
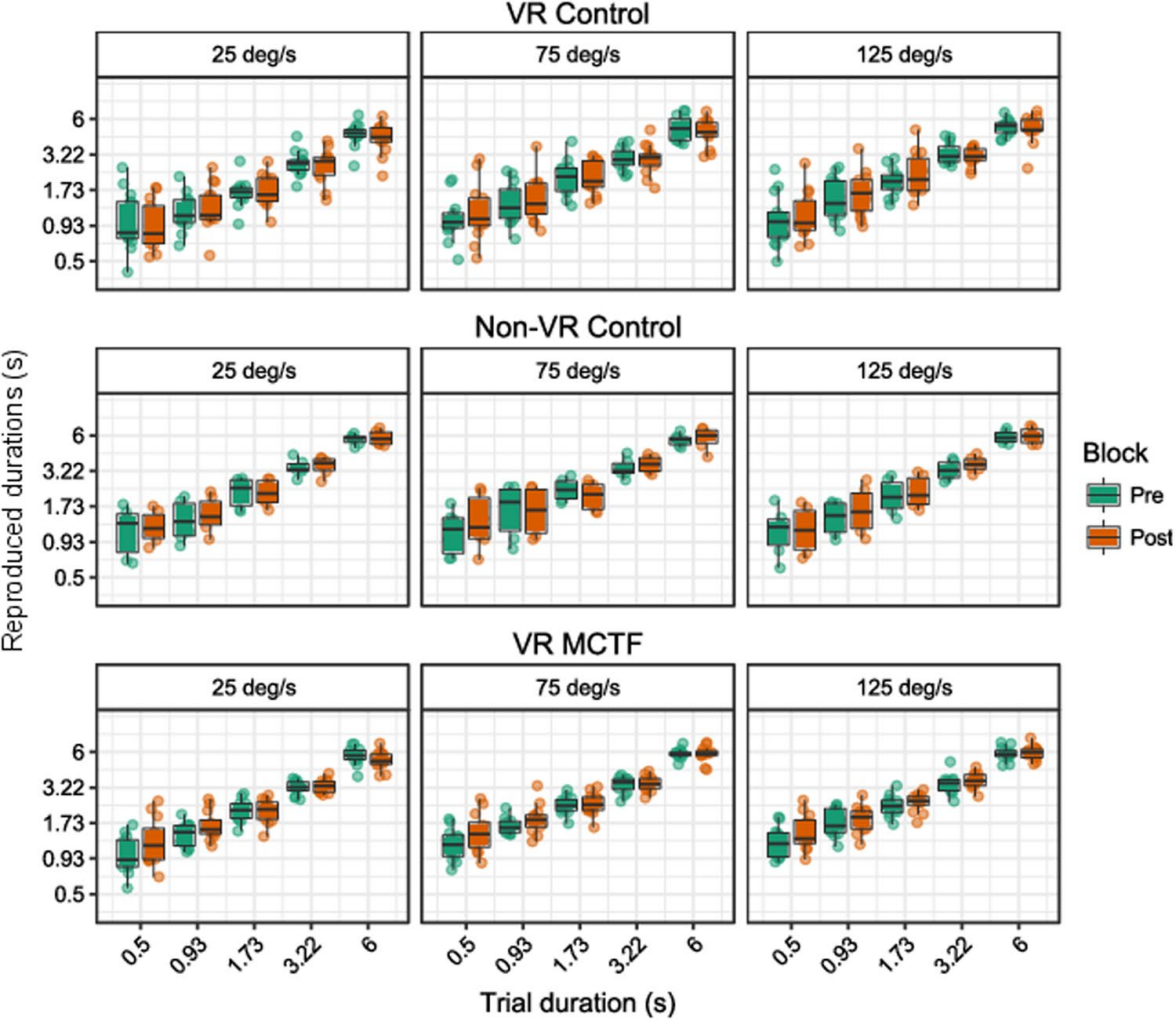
Tukey boxplots for duration estimates in the discrete motor time perception task, plotted on a log-log scale. Data are split by condition (VR control, non-VR control, VR MCTF), and trial velocity. Individual points are participant averages. Error bars indicate the farthest points within 1.5 x interquartile ranges^68^.

**Figure 5:**
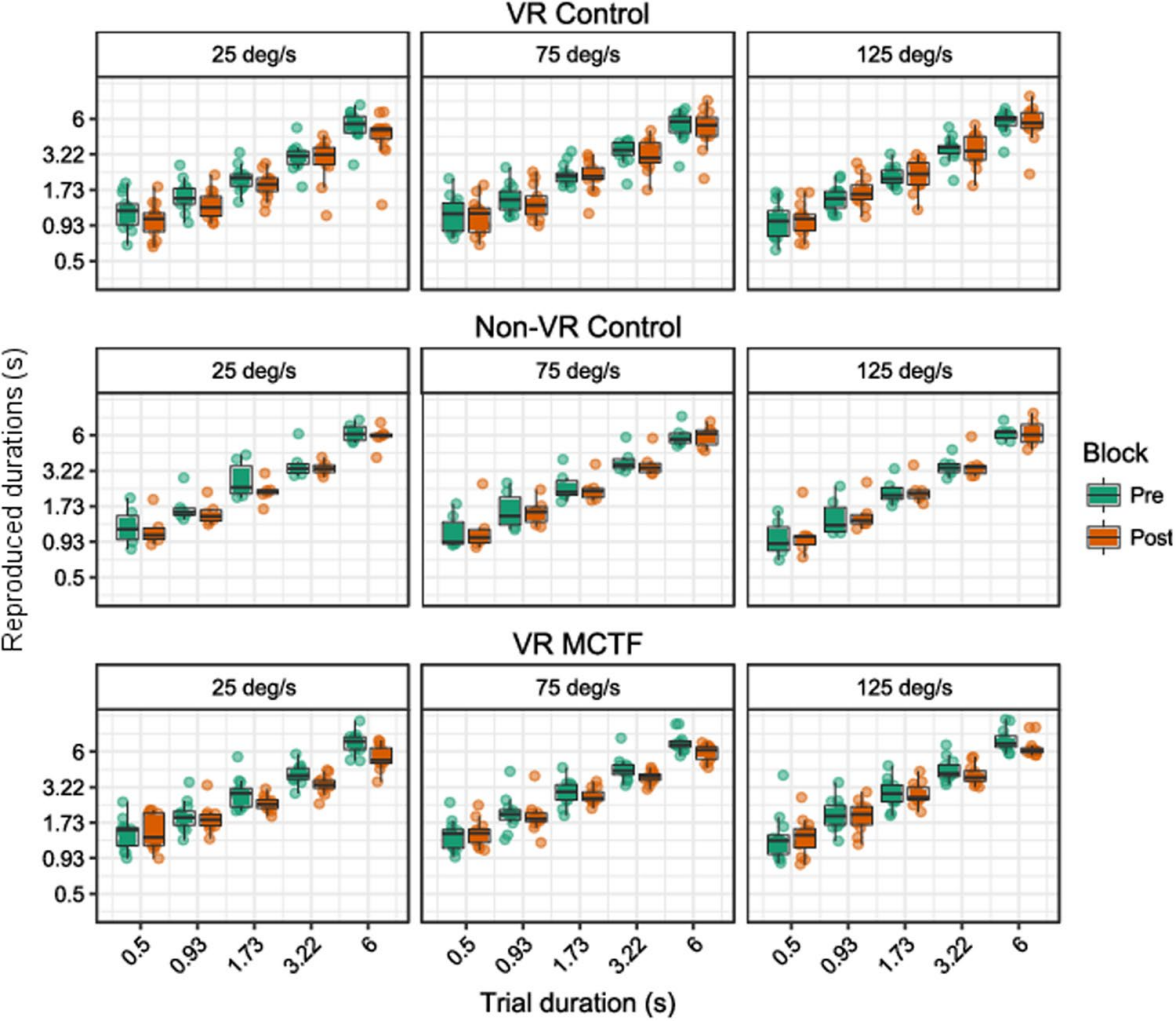
Tukey boxplots for duration estimates in the continuous motor time perception task, plotted on a log-log scale. Data are split by condition (VR control, non-VR control, VR MCTF), and trial velocity. Individual points are participant averages. Error bars indicate the farthest points within 1.5 x interquartile ranges.

**Figure 6:**
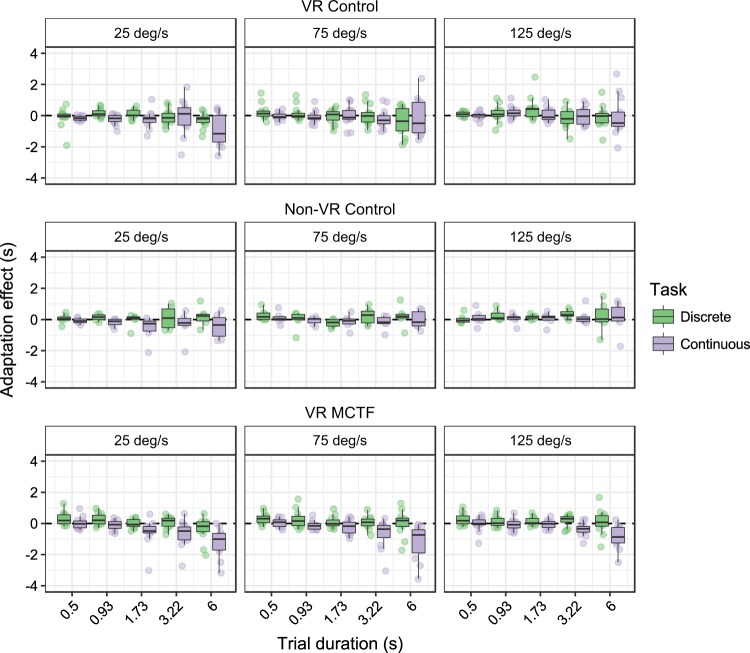
Conditions are as in the previous figure, but scores indicate adaptation effects (sec) by task type (continuous motor/discrete motor). Individual points are participant averages. Dotted line indicates zero adaptation. Error bars indicate the farthest points within 1.5 x interquartile ranges.

Raw scores for temporal duration estimates are plotted below for the discrete (Fig. 4) and continuous (Fig. 5) time perception tasks. These scores were separated by block and were used to compute ‘adaptation scores’: the difference in duration estimates between pre- and post-adaptation (see Fig. 6). As such, negative adaptation scores indicate that post-adaptation estimates were shorter than pre-adaptation estimates, and positive adaptation scores indicate that estimates were longer in the post-adaptation block.

We assessed differences between pre- and post-adaptation scores using a series of one-sample tests (Wilcoxon signed rank tests) on adaptation scores (post- minus pre-adaptation duration estimates) that were pooled across duration and velocity conditions. The results revealed adaptation effects that were significantly different from zero for the VR MCTF group in the continuous motor time perception task (*M* = −0.44 s, *SEM* = 0.13 s, Wilcoxon’s *V* = 3, *p* = 0.002), as shown in Fig. 6. For the VR MCTF group in the discrete time perception task (*M* = 0.12 s, *SEM* = 0.05 s, Wilcoxon’s *V* = 64, *p* = 0.052), and for the VR control group in both continuous (*M* = −0.12 s, *SEM* = 0.13 s, Wilcoxon’s *V* = 32, *p* = 0.38) and discrete (*M* = 0.01 s, *SEM* = 0.07 s, Wilcoxon’s *V* = 47, *p* = 0.95) tasks, we observed no evidence of significant adaptation.

To determine the source of the adaptation effects, we conducted a mixed design ANOVA with the within-subjects factors trial duration (0.5–6.0 sec), trial velocity (25/75/125**°**/s), task type (continuous/discrete motor time perception task), and the between-subjects factor MCTF (MCTF/normal time). Greenhouse-Geisser corrections were applied to account for sphericity assumption violations where appropriate.

Adaptation differed significantly as a function of both trial duration (*F*(1.63, 37.53) = 14.12, *p* < 0.001, Generalized Eta Squared (GES) = 0.10) and velocity (*F*(1.59, 36.52) = 10.95, *p* < 0.001, GES = 0.02). Trend analysis indicated that adaptation scores were more negative in the longer duration trials (linear trend *t*(92) = 6.93, *p* < 0.001, Cohen’s *d* = 0.72) and in the slower velocity trials (linear trend *t*(46) = 4.66, *p* < 0.001, Cohen’s *d* = 0.68). Task type also affected adaptation, with more negative adaptation scores in the continuous motor task than the discrete motor task (*t*(23) = 3.72, *p* = 0.001, Cohen’s *d* = 0.77). No main effect of VR MCTF was observed (*p* = 0.38).

### Interactions

There were several significant interactions, including interactions between MCTF and other factors that were relevant to our hypothesis. We observed a significant three-way interaction between MCTF, task type, and trial duration (*F*(2.04, 46.85) = 6.25, *p* = 0.004, GES = 0.02) and a significant two-way interaction between MCTF and task type (*F*(1, 23) = 5.58, *p* = 0.027, GES = 0.03). In addition, task type interacted significantly with both trial duration (*F*(2.04, 46.85) = 3.61, *p* = 0.015, GES = 0.01) and trial velocity (*F*(1.80, 41.37) = 3.75, *p* = 0.04, GES = 0.005). No other interactions were significant (*p*s ≥ 0.06).

### Post-hoc comparisons

We conducted least-squares means tests with Tukey adjustments for multiple comparisons^63^ to follow up significant interactions and main effects. Examining the three-way interaction, we found adaptation scores in the continuous motor task for the MCTF group were significantly more negative only at trial durations of 3.2 sec (*t*(127.04) = 2.51, *p* = 0.013, Cohen’s *d* = 0.22) and 6.0 sec (*t*(127.04) = 4.50, *p* < 0.001, Cohen’s *d* = 0.40) (other *p*s ≥ 0.23). For trials where the probe duration was 6.0 sec, the magnitude of adaptation was 0.87 sec, which indicates an increase in estimates of probe durations of 14.5%.

**Figure 7:**
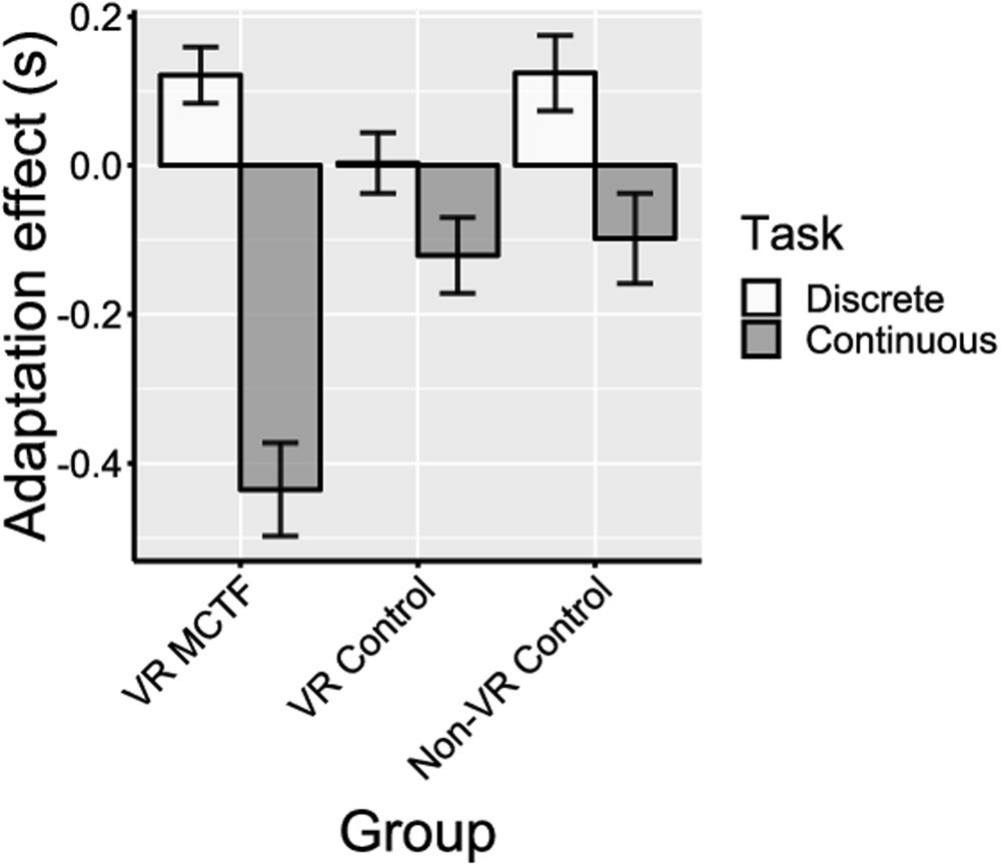
Adaptation effects for each group pooled across velocity and duration conditions. Negative adaptation effects indicate lower duration estimates post-adaptation compared to pre-adaptation. Error bars indicate *SEM*.

The interaction between MCTF and task type was due to adaptation scores being significantly more negative for the MCTF group in the continuous motor task (*t*(44.66) = 2.21, *p* = 0.03, Cohen’s *d* = 0.32) but not the discrete motor task (*p* = 0.41). This interaction is depicted in Fig. 7.

Next we examined the interaction between task type and duration/velocity of trials. Compared to the discrete motor task, adaptation scores in the continuous motor task were significantly more negative for all durations (*t*s(56.24) ≥ 2.17, *p*s ≤ 0.03, Cohen’s *d*s ≥ 0.28) except for 0.5 sec (*p* = 0.08). In the continuous motor task, adaptation scores tended to be more negative for low velocity trials than higher velocity trials (*t*s(88.62) ≥ 3.05, *p*s ≤ 0.06, Cohen’s *d*s ≥ 0.23; although the difference between 75 and 125**°**/s did not reach significance, *p* = 0.06). Velocity had no effect on adaptation scores in the discrete motor task (*p*s ≥ 0.15).

**Figure 8:**
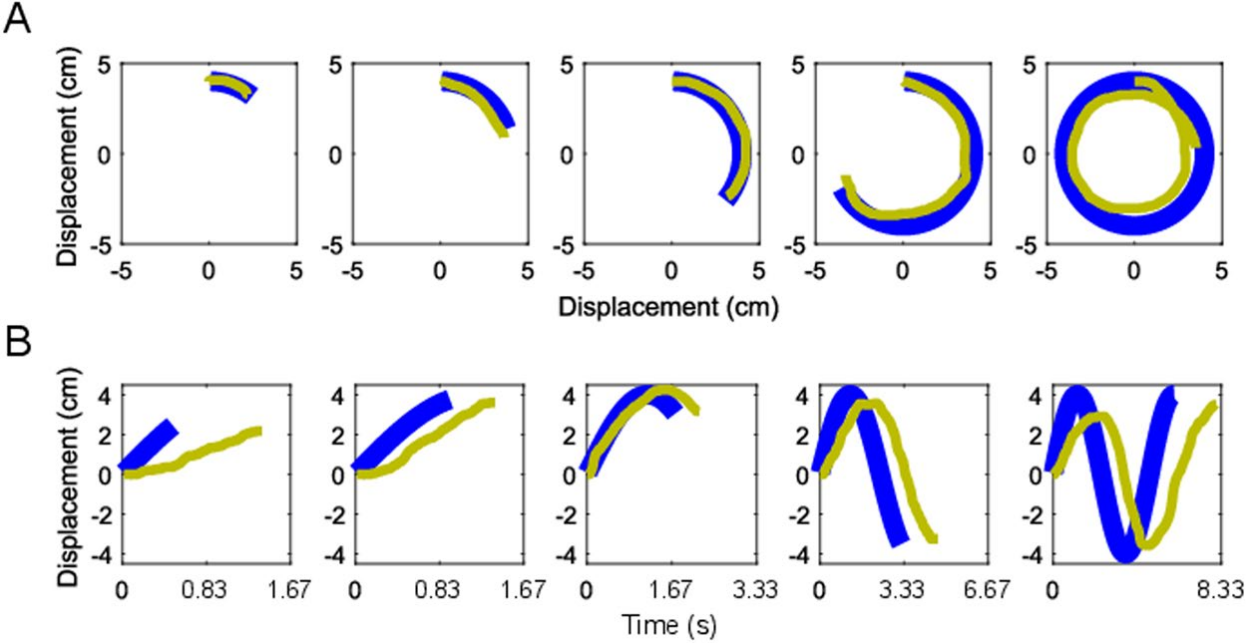
Exemplar trajectories for 5 trials from a single participant at the medium probe velocity (75**°**/s). Probe movement plotted in blue, participant reproduction plotted in yellow. (**A**) Participant and probe displacements in the X and Y-axis for the 5 durations (0.5 to 6.0 s). (**B**) Displacement of the probe and the participant’s reproduction over time in the X-axis of movement for the same trials. In these examples, the participant tended to over-estimate the probe duration, while maintaining a low spatial error in X and Y.

We also conducted a post-hoc analysis to assess if the difference in post-adaptation duration estimates between the VR MCTF and VR Control groups was attributable to group differences in spatial error when reproducing the movement of the probe. In order to assess spatial error we computed the absolute distance between the endpoint of the probe and the participant’s movement in each axis (left-right, and up-down; X and Y respectively) for each trial. We averaged these values across trials for each participant, resulting in average endpoint error estimates for X and Y. Exemplar spatiotemporal trajectories are plotted in Fig. 8.

We found no significant difference between the post-adaptation spatial error values for the VR MCTF and VR Control groups in terms of X (*t*(24) = 0.28, *p* = 0.78, Cohen’s *d* = 0.11), Y (*t*(24) = 0.30, *p* = 0.77, Cohen’s *d* = 0.12), or the average of the X and Y values (*t*(24) = 0.29, *p* = 0.77, Cohen’s *d* = *0*.10). The absolute spatial errors demonstrated by the two groups were as follows: VR MCTF, *M*_*X*_ = 12.46 mm, *SD*_*X*_ = 4.76 mm, *M*_*Y*_ = 12.57 mm, *SD*_*Y*_ = 7.36 mm; VR Control, *M*_*X*_ = 11.90 mm, *SD*_*X*_ = 5.34 mm, *M*_*Y*_ = 11.72 mm, *SD*_*Y*_ = 7.08 mm.

Given that the difference between duration estimates in the VR MCTF and VR Control groups was largest in the 6-second trials, we also assessed spatial errors in these trials alone. Again, no difference was observed between the groups with respect to X error (*t*(24) = 0.24, *p* = 0.81, Cohen’s *d* = 0.09), Y error (*t*(24) = 0.20, *p* = 0.84, Cohen’s *d* = 0.12), or averages of X and Y errors (*t*(24) = 0.24, *p* = 0.81, Cohen’s *d* = 0.09) in the 6 second probe trials (absolute spatial errors: VR MCTF, *M*_*X*_ = 15.03 mm, *SD*_*X*_ = 6.80 mm, *M*_*Y*_ = 13.83 mm, *SD*_*Y*_ = 9.15 mm; VR Control, *M*_*X*_ = 14.43 mm, *SD*_*X*_ = 5.99 mm, *M*_*Y*_ = 13.17 mm, *SD*_*Y*_ = 7.30 mm).

### Non-virtual reality control task

We conducted a similar analysis for data obtained in the non-VR control task. We assessed if there were adaptation effects using one-sample tests (Wilcoxon signed rank tests) on the adaptation scores. For both the continuous (*M* = −0.10 s, *SEM* = 0.14 s, Wilcoxon’s *V* = 8, *p* = 0.69) and discrete (*M* = 0.12 s, *SEM* = 0.11 s, Wilcoxon’s *V* = 16, *p* = 0.31) tasks, we found no evidence of significant adaptation. The results were overall highly similar to those obtained for the VR control group (see Fig. 6). As in the VR control group, we observed a significant effect of velocity on adaptation effects (*F*(2, 10) = 4.36, *p* = 0.043, GES = 0.05) which was driven by significantly more negative adaptation effects in the low velocity (25**°**/sec) trials compared to the high velocity (125**°**/sec) trials (*t*(19.87) = 3.22, *p* = 0.01, Cohen’s *d* = 0.70) only for the continuous motor task (discrete motor task *p*s ≥ 0.72). We observed no other significant main effects or interactions (*p*s ≥ 0.08).

### Cybersickness

Cybersickness levels were very low across participants. Only 5 participants reported cybersickness levels that were greater than zero, and of those 5 participants, the maximum score reported was 3 (range of 0 of 20), with an average of 2.38 (*SEM* = 0.26).

## Discussion

The plasticity of time perception at the sub-second scale is well established in previous literature^2–4,42^. Here we examined the potential for inducing a novel relationship between action and perception that would influence the perceived duration of time. In line with predictions, the results revealed significant effects of the manipulation we introduced in VR. These effects emerged only in the continuous motor time-perception task such that the probe intervals were under-estimated by approximately 15% following exposure to the VR MCTF manipulation for the longest duration trials. The lack of adaptation for VR and non-VR control groups supports the conclusion that temporal recalibration was induced by means of a novel sensorimotor contingency between movement and event speed.

The sensorimotor contingency theory of perception^49–51,64^ has been supported by evidence that novel relationships can be experimentally induced between perception and action for several perceptual qualia, including color^52^ and musical sound^65^. Consistent with these studies, our results indicate a significant reduction in the reproduced probe duration following adaptation to a movement-contingent VR game. The current results provide the first evidence for a novel contingency between movement and time perception. The findings have implications for our understanding of the embodied nature of temporal processing, and reiterate the potential for temporal recalibration that has been shown previously. This potential is highly relevant for future rehabilitation initiatives that focus on preventing or reversing maladaptive changes in temporal processing, such as those occurring with age.

The results revealed no evidence in support of temporal recalibration in participants who completed either the control VR task or control non-VR task. This supports a specific effect on the VR manipulation and suggests that neither VR nor physical activity alone were responsible for the temporal recalibration we observed. Adaptation effects were obtained in the continuous motor psychophysical task, but not the discrete motor task. We interpret this effect in terms of a sensorimotor contingency induced by coupling the speed of events in VR to the movement of the participant. In other words, participants who experienced the manipulation perceived the passage of time to be slower when they are not moving, and vice versa. This is indicated by a significant reduction in duration between the observation of the probe (static observer), and reproducing the movement (moving observer). In the discrete motor task, observing and reproducing the probe required no movement from the participant, and thus no effects of the novel sensorimotor contingency were observed.

Results revealed a significant reduction in the reproduced probe duration following exposure to the VR MCTF manipulation of approximately 400 ms on average. However, it should be noted that the majority of this effect was carried by significant adaptation for the longest duration trials, where approximately 0.9 sec adaptation was observed for 6 sec trials (~15% decrease in the reproduced probe duration). Note that while the MCTF manipulation caused event speed to slow by a factor of eight, the reproduced durations were only decreased by a factor of 1.15 in the condition that gave rise to the strongest effect here (6 sec trials). We interpret this result as evidence of a (partial) multiplicative slowdown in the perceived passage of time when the observer was stationary. For short trials (0.5 sec) a multiplicative slowdown would generate small differences between the perceived and reproduced durations, relative to the variability associated with internal/motor noise. At longer duration trials, however, a multiplicative modulation of the reproduced duration would result in larger effects that can be more easily detected among the noise sources. Since spatial error did not differ between the VR Control and VR MCTF groups (overall, and for only the 6 second trials), this leads us to rule out a specific effect of the MCTF manipulation on spatial errors during the reproduction task, and to instead conclude that temporal recalibration occurred for the VR MCTF participants.

Several previous studies have documented sub-second temporal recalibration. Vroomen and colleagues exposed observers to audio-visual stimuli that contained latencies of 100–200 ms^4^. Their results revealed a shift in the point of subjective simultaneity between two multisensory cues of approximately 10–15 ms that occurred in the direction of the exposure lag. Rohde and colleagues also reported sub-second temporal adaptation by introducing a 200 ms lag between hand and cursor movement in a manual tracking task^3^. The time course of adaptation to visuomotor latency revealed an adaptation effect in terms of a 30 ms decrease in motor error with time, and large aftereffects in motor timing. The effects we observed here were large relative to these previous studies, and this may be partially due to the exposure delays used in previous studies. For instance, Vroomen and co-workers^4^ did not expose participants to larger delays between multisensory cues than 200 ms due to the likelihood that greater delays would extinguish perceptual binding of the cues.

Participants were generally very reliable with respect to their responses on the time perception tasks, as we observed strong correlations between the reported and actual durations of the probes for the majority of participants. The results showed that participants tended to over-estimate durations for the shorter trials, especially at the shortest duration trials. This finding aligns with work by others showing overestimation of durations when short intervals (<1 sec) were reproduced^10,66,67^. However, the same studies identified trends consistent with underestimation for longer durations (5–10 sec), which we did not observe here. While the overestimation at short durations could indicate a perceptual effect (whose origin is unexplained), it might also reflect a methodological constraint on the speed at which participants could make a response. Future replications with more rapid response techniques (e.g., tactile interfaces) may shed light on this apparent overestimation.

In the current study, trial durations in the time perception tasks were limited to 6 seconds. Given that the short-duration trials typically revealed no adaptation effects, future replications of this study can benefit from discarding these conditions in favor of assessing whether similar effects emerge in longer duration trials. Another problematic issue inherent to our design was the lack of experimental control involved in the completion of the VR task. We employed an off-the-shelf, high fidelity VR game in our experiment to enhance the perceptual affordances and naturalism of the environment, but this choice meant that each participant had a slightly different experience while completing the task. This likely contributed a source of noise to the adaptation effects, given that some participants might have explored and engaged with the environment less than others. Greater experimental control is required to clarify the inter-individual variability in temporal recalibration effects. In addition, the movement of the participant was determined by the movement of the hands and the head, whereas in future experiments, a more compelling sensorimotor contingency may be induced if full body motion tracking is employed to modulate VR event speed. We also observed differences in the behaviour of participants through informal observations, but since we did not quantify this behaviour (e.g., analysis of head or hand velocity during VR), we are unable to determine if differences in performance strategies across participants in the VR MCTF group contributed to any variance in the adaptation effects. However, analyses of participant performance did not reveal any evidence of a difference in performance outcomes (i.e., total point scores). We intended the task to have a high ecological validity and strong affordances for action, and as such, the VR MCTF manipulation was expected to affect behaviour in overt and subtle ways. However, a full quantification of the behavioural changes produced by this manipulation would be a desirable outcome of future research.

According to the verbal self-report measures of comfort, participants experienced very little cybersickness during the experiment, suggesting that the VR task was a comfortable experience. These findings provide evidence that VR can be used to induce temporal recalibration, which may contribute to a clinical intervention in preventing or reversing the maladaptive changes to time perception, such as those observed in aging, Parkinson’s disease, schizophrenia, autism, and ADHD (for review, see^54^). However, the current findings are highly preliminary, and show an effect that is specific to a motor task involving continuous movement and may not be beneficial for rehabilitation purposes. Due to our specific time-flow manipulation, participants observed a reduction in the reproduced probe durations when stationary compared to when moving; in practice, it may be more useful to slow down the perceived passage of time when an individual is moving, thus enabling better control of the body (e.g., during a fall when establishing stable balance requires relocating the centre of gravity). It remains to be seen if adaptation effects opposite to those we obtained here can be produced. Several other extensions, refinements, and replications will be required before a similar paradigm is used in a clinical setting.

How does the brain represent the temporal recalibration effects observed here? Although our experiment was not designed to test mechanistic theories of time perception, our findings are consistent with multiple accounts, including the internal clock^11^. For instance, a movement-dependent increase in the number of pulses emitted by the internal oscillator per unit of time, or modulation of the gating of these pulses by the calibration unit (as in the click train effect^2,14,42^, would result in the pattern of data obtained. At the same time, the spatial or temporal pattern of neuronal activity may have been modulated by adaptation^17–19^. The transfer of adaptation from a VR setting to a simple psychophysics task supports adaptation of a central mechanism for time perception, in line with other evidence^13^. However, future neuroimaging studies are needed to provide insight into the neural basis of sensorimotor contingency acquisition for temporal processing.

In summary, here we exposed participants to a VR experience where movement and event-speed were experimentally coupled, and we observed evidence that a novel relationship between action and event-speed caused recalibration of time perception in a psychophysical task. The sensorimotor contingency between movement and the speed of events induced temporal recalibration that did not emerge in control VR and non-VR conditions. This study provides further evidence of the flexibility of time perception, and indicates that sensorimotor contingency theory offers a useful framework for studying high-level perceptual qualia such as time perception. The utility of VR for modulating time perception is also evident from these results, although future refinements are needed before the practical relevance of these findings can be established.

## Supplementary information

Supplementary Movie 1

## Data Availability

The data that support the findings of this study will be made freely available upon publication on the Open Science Framework: Bansal, A., Weech, S., & Barnett-Cowan, M. (2018, September 10). Movement-Contingent Time Flow in Virtual Reality Causes Temporal Recalibration. Retrieved from osf.io/nt2jh.
